# Foraging behaviour of a continental shelf marine predator, the grey seal (*Halichoerus grypus*), is associated with in situ, subsurface oceanographic conditions

**DOI:** 10.1186/s40462-020-00225-7

**Published:** 2020-10-20

**Authors:** B. V. R. Nowak, W. D. Bowen, K. Whoriskey, D. C. Lidgard, J. E. Mills Flemming, S. J. Iverson

**Affiliations:** 1 Department of Biology, Dalhousie University, Halifax, Nova Scotia B3H 4JI Canada; 2 Population Ecology Division, Department of Fisheries and Oceans, Bedford Institute of Oceanography, Dartmouth, Nova Scotia B2Y 4A2 Canada; 3 Department of Mathematics and Statistics, Dalhousie University, Halifax, Nova Scotia B3H 4JI Canada

**Keywords:** Scotian Shelf, Grey seal, Hidden Markov model, Foraging behaviour, Benthic, Oceanographic conditions, Phytoplankton biomass

## Abstract

**Background:**

The heterogeneous oceanographic conditions of continental shelf ecosystems result in a three-dimensionally patchy distribution of prey available to upper-trophic level predators. The association of bio-physical conditions with movement patterns of large marine predators has been demonstrated in diverse taxa. However, obtaining subsurface data that are spatio-temporally relevant to the decisions made by benthically-foraging species can be challenging.

**Methods:**

Between 2009 and 2015, grey seals were captured on Sable Island, Nova Scotia, Canada during summer and fall and instrumented with high-resolution archival GPS tags. These tags recorded location data as well as depth (m), temperature (°C), and light level measurements during dives, until animals returned to the haulout site to breed. Hidden Markov models were used to predict apparent foraging along movement tracks for 79 individuals (59 females, 20 males) every 3 h. In situ measurements were used to estimate chlorophyll-*a* concentration (mg m^− 3^) and temperature within the upper-water column (50 m) and temperature and depth at the bottom of dives. As chlorophyll-*a* could only be estimated from 10:00 to 14:00 AST for dive depths ≥50 m, we formulated two generalized linear mixed-effects models to test the association of predicted grey seal behavioural states with oceanographic conditions and phytoplankton biomass: the first representing conditions of the upper-water column likely to influence primary productivity, and a second model including environmental conditions encountered by grey seals at the bottom of dives, when seals were more likely to be foraging.

**Results:**

Predicted grey seal behavioural states were associated with fine-scale chlorophyll-*a* concentrations and other environmental conditions they encountered across the continental shelf. In the Water Column Model, season had no influence on the probability of observing apparent foraging, but chlorophyll-*a*, upper-water column temperature, and sex did, with females having a greater probability of foraging than males. In the Bottom Conditions Model, again season had no influence on the probability of apparent foraging, but females were over twice as likely as males to be foraging.

**Conclusions:**

The results of this study highlight the value of in situ measurements of oceanographic properties that can be collected at high temporal resolution by animal-borne data loggers. These data provide insight into how inferred behavioural decisions made by large marine predators, such as the grey seal, may be influenced by fine-scale oceanographic conditions.

## Background

Physical and biological oceanographic features of continental shelf ecosystems are dynamic over a range of spatio-temporal scales [1]. This results in some areas having disproportionately high levels of primary productivity [2] that support assemblages of species at higher trophic levels [3]. The distributions of fish and invertebrate species are constrained by a suite of preferred environmental conditions (e.g., temperature, depth, salinity), in addition to food availability [4]. The heterogeneous nature of oceanographic conditions results in a three-dimensionally patchy distribution of prey available to upper-trophic level predators [5]. Where prey are concentrated (i.e., within patches), foraging success should be higher as less time and energy is expended searching and thus the quantity of prey that can be consumed is likely to be higher [6]. This patchiness can persist throughout the food web, exerting bottom-up control on local species abundances, and result in multi-trophic level hotspots [5, 7].

Oceanographic conditions have been linked to the movements and foraging patterns in diverse marine taxa [5], including sea turtles [8], fishes [9], seabirds [10], and marine mammals [11, 12]. These studies often use oceanographic data derived from remote sensing [13] and a knowledge of persistent, predictable meso-scale (10s – 100s km) bio-physical features (i.e., topography, fronts, or current systems) [12, 14, 15]. Broad-scale spatial associations with oceanographic features (e.g., proximity to eddies [16]) and characteristics of diving behaviour within them [17, 18] have been used to infer foraging behaviour. Nevertheless, how these features influence foraging behaviour at finer scales remains unclear [19].

An alternative approach is to relate oceanographic conditions encountered by predators to inferred behavioural states using estimated prey encounters [20], state-space models [21], or hidden Markov models (HMMs) [22]. HMMs have become particularly popular, as accurate location data become increasingly available, due to their flexibility, speed, and intuitive results [23]. The ability to infer multiple at-sea behaviours, such as “travelling” and “apparent foraging” (i.e., area-restricted search), from animal movement data allows for a better understanding of the intrinsic and extrinsic drivers of movement patterns [24].

The suite of environmental conditions encountered by foraging individuals may influence foraging decisions either directly, or indirectly by structuring the distribution of prey. This may be particularly true where bio-physical features are highly dynamic or occur at fine scales [25]. Obtaining concurrent oceanographic and animal movement data at scales relevant to foraging decisions remains challenging [26]. Although remotely sensed oceanographic data have proven useful for pelagic species that dive during foraging but otherwise remain near-surface [27], they are less useful for species that both forage and travel near the ocean floor, where prey are likely to be influenced by conditions at-depth. To overcome these challenges, there has been growing interest in using large marine predators to collect oceanographic data along their movement tracks, particularly in polar regions where pinnipeds are abundant and satellite coverage is high [28]. These data can be used together with movement characteristics to improve our understanding of how oceanographic conditions influence behaviour [29, 30].

The grey seal (*Halichoerus grypus*) is a relatively large-bodied phocid species inhabiting mid-latitude continental shelves on both sides of the North Atlantic Ocean. The western North Atlantic population is large and increasing [31], with Sable Island being the location of the largest breeding colony worldwide [32]. Seals from this colony make foraging trips, spatially segregated by sex and season, throughout the Scotian Shelf (SS) ecosystem [33, 34]. Although foraging is concentrated over offshore banks [33], the bio-physical processes (e.g., circulation, temperature) that surround these banks may change over time. Fish species consumed by grey seals exhibit seasonal variation in spatial distributions across the SS (e.g., [35]). We hypothesize that oceanographic conditions therefore have a high potential of influencing, either directly or indirectly, foraging patterns exhibited by grey seals.

The SS is topographically complex with a series of banks and basins largely concentrated over the eastern Scotian Shelf. These features influence the hydrodynamic properties of the region, as cooler, fresher water from the Gulf of St. Lawrence becomes coastally-trapped as the Nova Scotia Current and permeates across the eastern Scotian Shelf to form the top layer of this stratified shelf sea [36, 37]. Inflow of warmer, more saline waters from the slope occurs through deeper channels such as the Gully, but due to density gradients are largely unable to flow above the shallow banks [38]. This results in distinct bottom climatologies that have been used to differentiate the eastern Scotian Shelf from the central and western Scotian Shelf subregions [39]. Together, these features result in fine-scale circulation patterns that vary three-dimensionally across the continental shelf [40, 41].

Here we examine the association of grey seal behavioural states inferred from an HMM with oceanographic conditions using environmental data collected in situ by grey seals. As previous studies have shown strong sex-specific and seasonal differences in ranging [33], foraging behaviour [42], and diet [43] of grey seals in our study population, we tested hypotheses that the association of oceanographic conditions with estimated behaviours may differ by sex and season.

## Methods

The study was conducted on Sable Island (43°57′N, 59°55′W), a crescent-shaped sandbar located on the eastern Scotian Shelf approximately 300 km east of Halifax, Nova Scotia, Canada. One-hundred-seventeen adult grey seals (83 females, 34 males) were instrumented with telemetry and biologging devices (Table 1). Individuals were captured onshore in summer following the spring moult (June) or fall (late September or early October) using handheld nets. They were then immobilized with an intramuscular injection of Telazol (female dose 0.90 mg kg^− 1^, male dose 0.45 mg kg^− 1^). Standard body length and body mass were recorded. Each seal was equipped with an archival Mk10-AF Fastloc™ GPS bio-logging device (time-depth-light recorder, TDLR; Wildlife Computers, www.wildlifecomputers.com), which must be recovered, and a VHF transmitter (164 to 165 MHz; www.astrack.com), to permit relocation in the breeding colony and recapture the following December/January. The VHF transmitter was attached to the TDLR using a stainless-steel hose clamp and both were glued to the fur on the top of the seal’s head using 5 min epoxy. Tags recorded temperature (°C), depth (m), light level (*LL*), and condition (wet/dry) every 10 s during dives and Fastloc GPS locations after every 15 min when the animal was at the surface. GPS locations were suspended during haul out periods once a location had been recorded and the tag detected dry conditions for 45 s out of every 1 min for 20 min. Location attempts resumed when the seal returned to sea and the tag detected wet conditions for 45 s in 1 min. GPS locations derived from < 5 satellites and/or residual error values > 30 were removed from the data [44, 45]. A speed filter of 10 m s^− 1^ was also applied to remove erroneous locations. The remaining locations were considered to have negligible error and accuracies of 10s of meters [46]. Temperature was measured using a fast-response external thermistor within a range of − 40 to 60 °C at a resolution of 0.05 ± 0.1 °C. Depth was measured between 0 and 1000 m with a resolution of 0.5 m and an accuracy of 1% of the depth reading. Light sensors were comprised of a photodiode with a blue-window transmittance filter resulting in a peak sensitivity of 465 nm and parabolic range between 400 and 490 nm [47]. Light intensity was detected between 5 × 10^− 12^ W cm^− 2^ and 5 × 10^− 2^ W cm^− 2^ and log-transformed onboard tags to a three-digit *LL* value, resulting in a range of 25 to 225 units.

**Table 1:** Number of deployments and recoveries of Mk10-AF Fastloc™ GPS time-depth-light recorders from grey seals on Sable Island, NS by year, season, and sex

Year	Deployment Month	Instruments Deployed	Instruments Recovered	Data Recovered		
				Total	Males	Females
2009	October	15	13	13	5	8
2010	September	20	20	20	6	14
2011	June	20	16	13	0	13
2012	June	17	16	15	5	10
2013	June	15	12	12	4	8
2014	June	15	12	12	5	7
2015	June	15	11	9	0	9
Total		117	100	94	25	69

Dive data were analysed using WC-DAP, freely available software provided by the tag manufacturer. Dives shallower than 5 m were removed from the dataset to reduce the influence of surface conditions (e.g., wave action) and near-surface rolling [42]. Those >30 min were also removed to avoid misidentification of consecutive dives merged together by dive analysis software [42]. Data were automatically zero-offset corrected to account for pressure transducer shift onboard tags and dives were separated into three phases (i.e., descent, bottom, ascent). Summary statistics for each dive included duration, descent rate, bottom duration, ascent rate, and maximum depth. Bottom duration was defined as the time spent at depths ≥80% of the maximum depth for each dive, standard to the dive analysis software. Dives were then filtered using R [48] by removing those with ascent and descent rates ≥6 m s^− 1^ or equal to 0 m s^− 1^ [49] as well as those ≤20 s to remove those that were biologically impossible and surface behaviours that were misidentified as dives.

### Environmental data

Recorded environmental data were assigned to a dive and phase using a purpose-built algorithm. The ascent phase of dives was used to calculate the mixed-layer depth (m), mean upper-water column temperature (*T*_*50*_; °C), and light attenuation (*LA*; m^− 1^) within the upper-water column (50 m) for each dive. A depth of 50 m includes most mixed-layer depths in our study area and the majority of the phytoplankton biomass [50, 51]. *LL* measurements were linearly regressed over the upper-water column to estimate *LA*. *LA* data were then used to calculate chlorophyll-*a* concentration (chl-*a*; mg m^− 3^) using a locally-validated bio-optical model [50] and were restricted to a 4 h period surrounding local noon (10:00 to 14:00 AST) to reduce the influence of solar zenith angles [52]. Mean dive depth (m) and temperature (°C) were calculated for the bottom phase of each dive to describe the environmental conditions encountered by grey seals, which primarily forage benthically [53, 54].

### Hidden Markov model

HMMs can be used to predict discrete mathematical states along movement tracks that can then be interpreted as putative animal behaviour. Hereafter we will refer to these human-inferred behaviours as behavioural states. We fitted an HMM (using the R package *swim* version 0.2.4; https://github.com/kimwhoriskey/swim/) to predict behavioural states for individual grey seal movement tracks [55]. With this HMM, the movement of an animal is modelled as a discrete-time correlated random walk on the displacement between successive locations (e.g., the first-difference correlated random walk or DCRW of [56]). The parameters governing the movement process include a turning angle (θ) and an autocorrelation in both direction and speed (γ). We fitted a two-state HMM, and therefore allowed θ and γ to each take on one of two values dependent on the state. Typically, directed movement is achieved by a low turning angle and high amount of autocorrelation (θ ≈ 0 and γ > 0.5), while tortuous movement is characterized by a high turning angle and low amount of autocorrelation (θ ≈ π and γ <  0.5). We interpret these as “travelling” and “apparent foraging” behavioural states, respectively.

Archived Fastloc GPS location data were highly accurate, collected at a high sampling frequency, and stored onboard tags [57]. Movement tracks were visually assessed for temporal gaps prior to HMM analysis. As the HMM is a discrete-time model, we interpolated along the tracks using a time step chosen prior to the HMM analysis. We chose 3 h, given distances to foraging patches, foraging patch sizes and residence times, and swim speeds when in the apparent foraging state [33]. Interpolation can introduce error in the observed locations when temporal gaps larger than the time step are present. However, given our coarse time step (i.e., 3 h) relative to the tag transmission times (i.e., 15 min) and lack of temporal gaps in at-sea locations, we are confident that this error was small. While some studies incorporate environmental covariates into the transition probabilities (e.g., [58]), we were unable to because one of our covariates, chl-*a*, could only be estimated for 4 h each day, and together with upper-water column temperature, if dives reached 50 m depth.

### Statistical analysis

To examine whether predicted behavioural states were associated with environmental conditions encountered by grey seals during foraging trips, we fit generalized linear mixed-effects models (GLMMs), allowing for the analysis of non-Normal data and inclusion of individual as a random effect. In our case, the random effect was included to account for the fact that these individuals were randomly selected from the larger population, and although inter-individual heterogeneity was not of direct interest, it should be controlled for. As our response variable was a realization of a first-order Markov chain, temporally-adjacent values were autocorrelated. To account for this correlation, as well as gaps present in the data when environmental data were not available (i.e., animals were hauled out) or when chl*-a* estimates could not be made, we specified a continuous first-order autoregressive structure CAR (1). Season was included as a categorical variable and assigned as summer (June–August) and fall (September–December) [33]. Median values of environmental conditions leading up to each location associated with a behavioural state were taken to be representative of conditions encountered by grey seals during decision making. Because chl-*a* could only be estimated from 10:00 to 14:00 AST at dive depths ≥50 m [50], we formulated two models: the first representing conditions of the upper-water column likely to influence productivity, and a second model including environmental conditions encountered by grey seals at the bottom of dives, when seals were more likely to be foraging. The full models, including main effects and two-way interaction terms were:

*Water Column Model (Model 1):*

$$ {\displaystyle \begin{array}{l} logit\left({p}_{i,t}\right)={\eta}_{i,t}\\ {}{\eta}_{i,t}={\beta}_0+{\beta}_{1a}{chl}_{i,t}+{\beta}_2{T}_{50,i,t}+{\beta}_3{sex}_i+{\beta}_4{season}_t+{\beta}_5{chl}_{i,t}\ast {sex}_i+{\beta}_6{chl}_{i,t}\ast {season}_t+{\beta}_7{T}_{50,i,t}\ast {sex}_i+{\beta}_8{T}_{50,i,t}\ast {season}_t+{\beta}_9{sex}_i\ast {season}_t+{\nu}_{seal}+{\epsilon}_{i,t}\end{array}} $$

*Bottom Conditions Model (Model 2):*

$$ {\displaystyle \begin{array}{l} logit\left({p}_{i,t}\right)={\eta}_{i,t}\\ {}{\eta}_{i,t}={\beta}_0+{\beta}_1{dur}_{i,t}+{\beta}_2{T}_{i,t}+{\beta}_3{depth}_{i,t}+{\beta}_4s{ex}_i+{\beta}_5{season}_t+{\beta}_6{dur}_{i,t}\ast {sex}_i+{\beta}_7{dur}_{i,t}\ast {season}_t+{\beta}_8{T}_{i,t}\ast {sex}_i+{\beta}_9{T}_{i,t}\ast {season}_t+{\beta}_{10}{depth}_{i,t}\ast {sex}_i+{\beta}_{11}{depth}_{i,t}\ast {season}_t+{\beta}_{12}{sex}_i\ast {season}_t+{\nu}_{seal}+{\epsilon}_{i,t}\end{array}} $$

where *p*_*i,t*_ corresponds to the probability of observing apparent foraging for the behavioural state for individual deployment *i* at time *t*, *η*_*i*, *t*_ is the corresponding linear predictor, *chl*_*i*, *t*_ is estimated chl-*a*, *T*_50, *i*, *t*_ is the mean temperature of the upper-water column, *ν*_*seal*_ denotes the random effect of individual seals with autocorrelated structure in the covariance matrix, *ϵ*_*i*, *t*_ describes the random deviation in the model independent of *ν*_*seal*_, *dur*_*i*, *t*_ is the bottom duration, *T*_*i*, *t*_ is the mean bottom temperature, and *depth*_*i*, *t*_ is the mean dive depth. Models were fitted using penalized quasi-likelihood estimation with the function *glmmPQL* in the R package *MASS* [59, 60]. This software allows for a binomial response to accommodate behavioural states 0 (travelling) and 1 (apparent foraging), inclusion of a random effect, and specification of an appropriate residual autocorrelation structure CAR (1). The computation of quasi-likelihoods meant that these models were not suitable for model comparison and we were limited to hypothesis testing of the *t* test statistics produced by model outputs. For our analysis, we were concerned with quantitative parameter estimates, which were transformed to odds ratios for interpretation. Assumptions of the GLMMs included (i) independence, (ii) absence of multicollinearity, and (iii) linearity of continuous independent variables with data transformed by the link function. Depth was log-transformed to meet the assumptions of the GLMM and can be seen to have a linear relationship with the response. The inclusion of the autocorrelation structure improved fitted models compared to models that did not include the structure, with little to no residual autocorrelation present. Model diagnostics included graphical checking of residuals and assessment of the random effect estimates and variance.

## Results

Data stored onboard tags were successfully recovered from 94 individuals (69 females and 25 males, Table 1). Age, body mass, and standard body length of instrumented seals are given in S1. Seventeen seals did not return to Sable Island to breed and data (e.g., GPS location, temperature, or depth) from another six seals contained too many errors to reliably reconstruct movements or oceanographic data. In 2012, wet/dry sensors intermittently malfunctioned on all tags resulting in fewer GPS locations and large temporal gaps. Therefore, data collected in 2012 were omitted from HMMs, with data from 79 individuals (59 females and 20 males) being included in GLMMs.

**Fig. 1:**
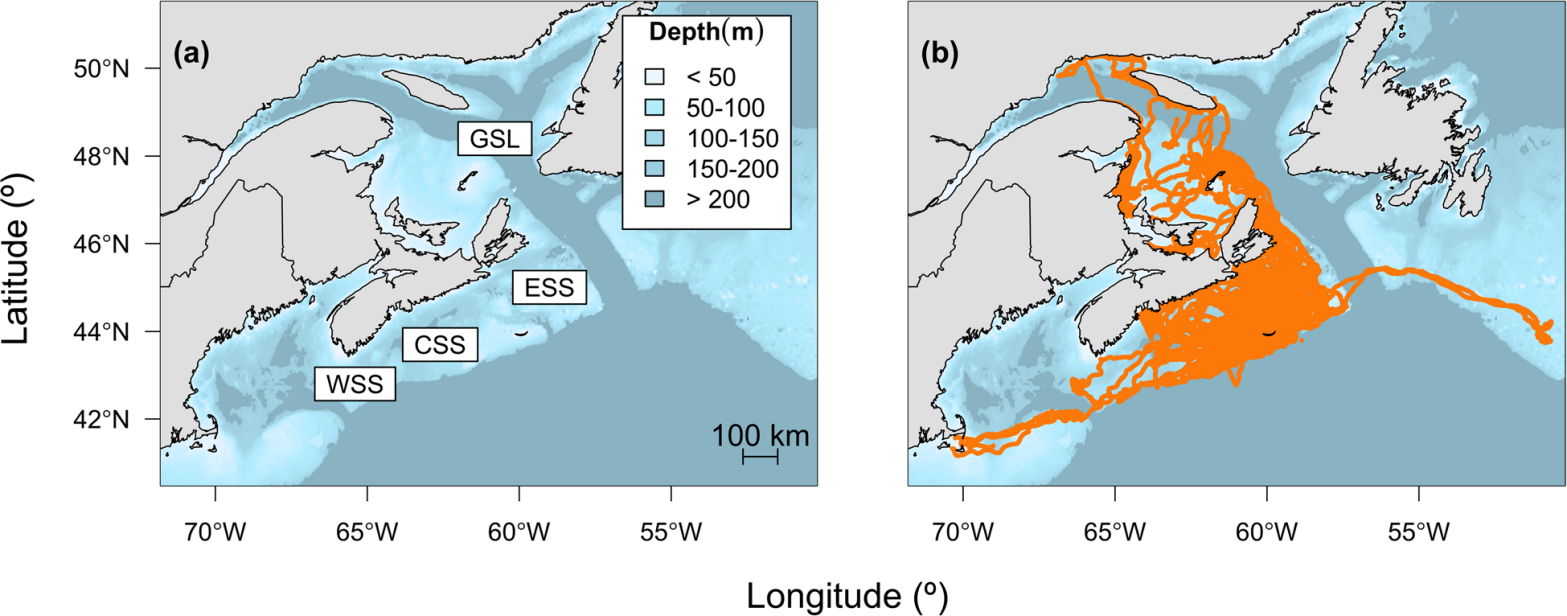
**a** Scotian Shelf ecosystem with the eastern Scotian Shelf (ESS), central Scotian Shelf (CSS), western Scotian Shelf (WSS), and Gulf of St. Lawrence (GSL) subregions identified and **b** spatial distribution of grey seal (*n* = 79) locations obtained between June and December over the study period

**Fig. 2:**
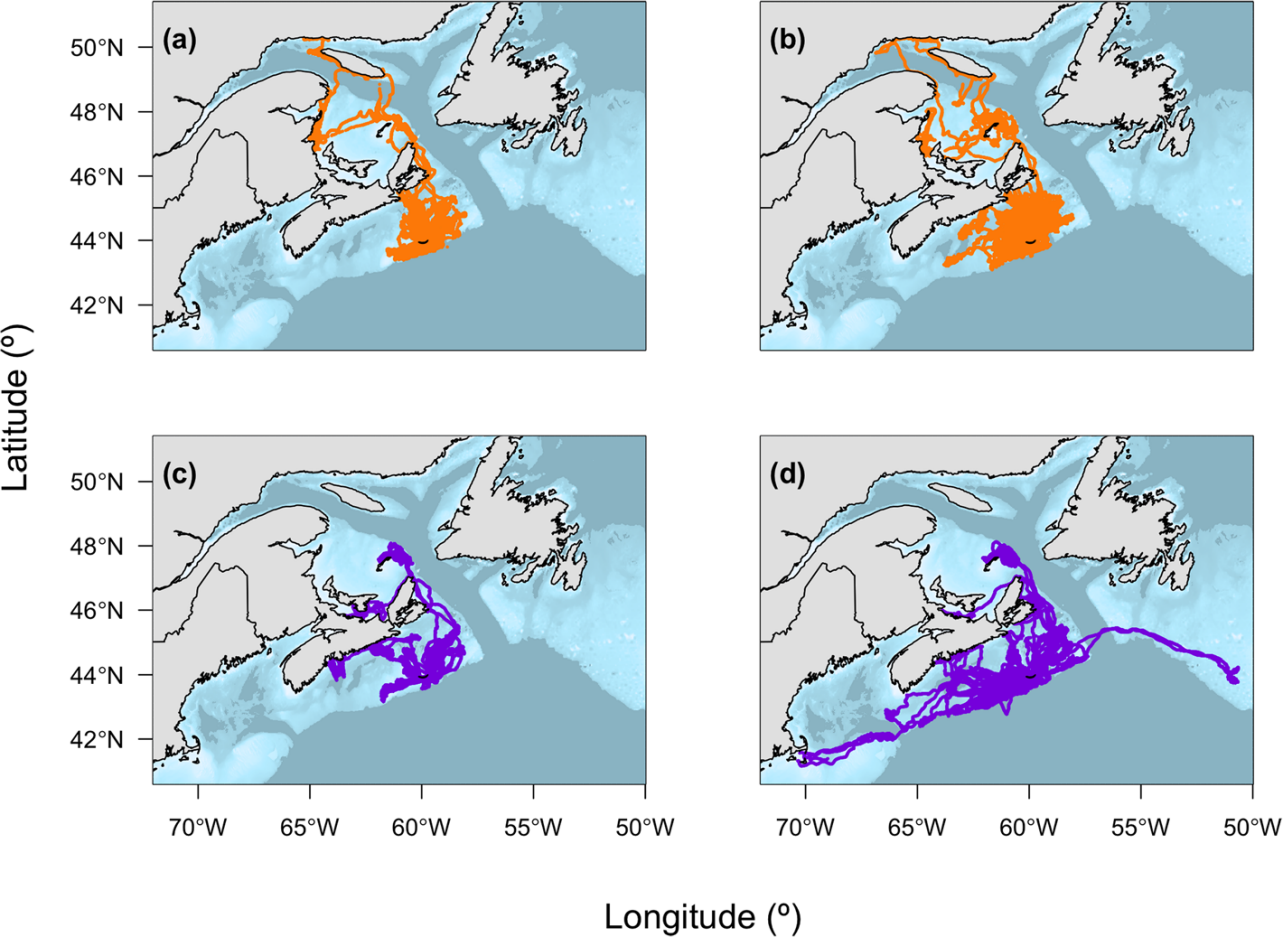
Locations of grey seals (*n* = 79) between June and December over the study period separated by season and by sex: **a** females in summer (*n* = 37), **b** females in fall (*n* = 59), **c** males in summer (*n* = 9), and **d** males in fall (*n* = 20)

**Fig. 3:**
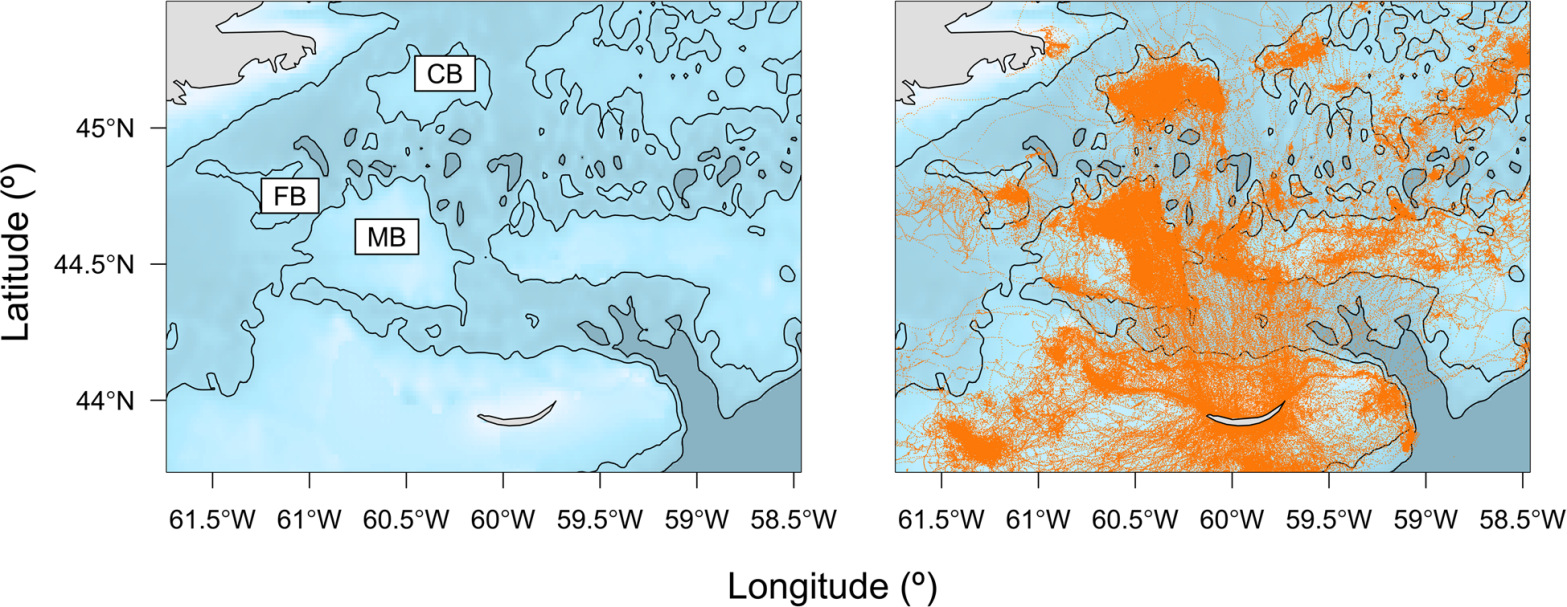
Locations of instrumented grey seals between June and December throughout the study period (*n* = 79) to highlight fine-scale habitat use over offshore topographical features, such as Middle Bank (MB), Canso Bank (CB), and French Bank (FB). Isobaths at 100 m and 200 m depths are included as black lines

A total of 1,668,086 dives and 569,349 locations were recorded from 79 individuals (Table 2). Few locations were available in January as adults returned to the breeding colony at Sable Island. Therefore, January data were excluded (also see [33]). Individuals were tracked for an average of 180 days, except during 2009 and 2010 when deployments occurred in the fall (Table 2). The combined-sex spatial distribution of at-sea locations shows frequent use by grey seals of the central and eastern areas of the SS and parts of the Gulf of St. Lawrence (Fig. 1). Although the spatial distributions of males and females largely overlap during the summer, males tended to range farther than females on the SS during the fall (Fig. 2), as previously reported [34]. GPS location data also revealed fine-scale habitat use over shallow topographical features, such as Middle and Canso Banks (Fig. 3) that was not evident in earlier studies using less accurate Argos locations. For example, grey seals showed disproportionately high use of the eastern side of Middle Bank compared to the western side, whereas the whole of Canso Bank was heavily used by grey seals.

**Table 2:** Sample means and standard deviations (SD) of the duration of deployment (days), number of dives, duration of time spent diving (days), proportion of time spent diving, number of locations, resulting number of hidden Markov model (HMM) locations at the three hour time step, and proportion of HMM locations spent foraging (*n* = 79)

	2009		2010		2011	2013		2014		2015
	M	F	M	F	F	M	F	M	F	F
**Duration**	73.21	66.1	100.1	102.4	183.3	180.6	182.8	194.0	194.6	192.6
SD	1.05	3.96	7.83	6.21	20.99	6.75	6.00	6.98	7.80	7.50
**Dives**	11,925.0	10,909.6	13,707.5	15,900.6	24,137.9	31,052.3	25,136.6	28,972.4	27,305.0	26,804.2
SD	1206.85	1223.22	1212.17	2772.25	4314.94	5088.26	4756.96	3502.61	4468.37	3315.80
**Dive Time**	43.0	44.1	59.7	67.2	111.1	109.6	114.1	110.5	118.9	111.7
SD	4.62	4.18	3.56	6.44	16.40	7.02	8.53	8.72	8.83	12.66
**Dive Proportion**	0.59	0.67	0.60	0.66	0.61	0.61	0.62	0.57	0.61	0.58
SD	0.07	0.04	0.06	0.04	0.05	0.05	0.04	0.04	0.04	0.06
**Locations**	7681.6	4150.5	4776.7	5532.1	6528.9	8683.3	7954.6	10,412.2	10,503.6	9199.4
SD	3271.06	352.02	1583.48	1484.88	2622.60	3804.74	2086.70	1012.52	1178.81	981.29
**HMM Locations**	494.2	462.7	641.5	684.1	1119.7	1199.3	1144.7	1197.2	1225.4	1163.2
SD	19.04	33.04	71.15	48.28	153.65	86.09	78.03	82.67	81.34	106.26
**Foraging Proportion**	0.47	0.58	0.60	0.77	0.74	0.49	0.72	0.72	0.83	0.78
SD	0.17	0.33	0.18	0.07	0.12	0.21	0.23	0.12	0.07	0.04

**Fig. 4:**
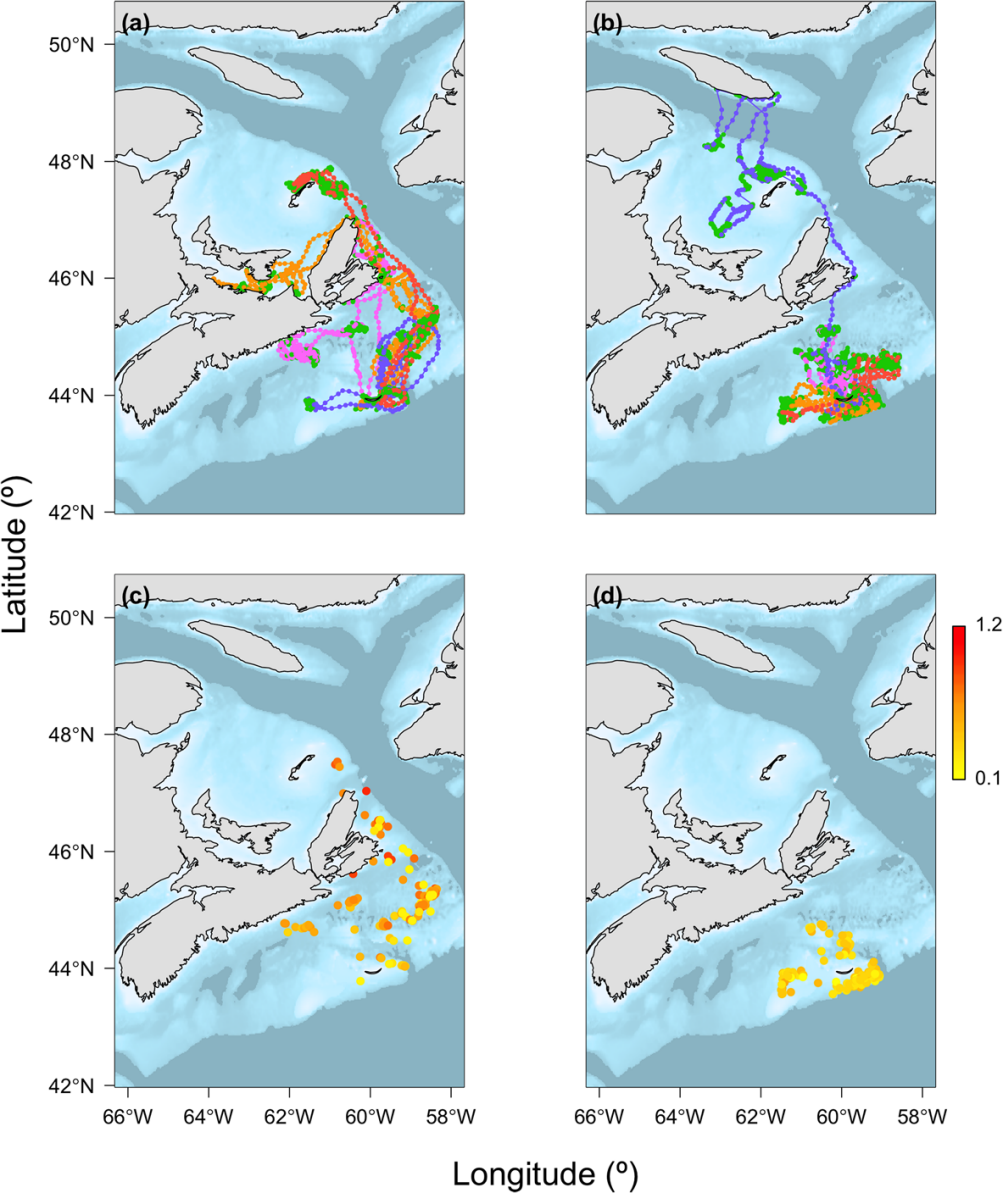
Top: Examples of interpolated locations and corresponding behavioural states (green = apparent foraging and other unique colours = travelling by individual seals) predicted by the hidden Markov models for **a** four males and **b** four females. Bottom: Estimated chlorophyll-*a* concentrations (mg m^− 3^) for those **c** males and **d** females

Oceanographic data were assigned to 73,144 interpolated locations along with corresponding behavioural state estimates produced by HMMs (Table 2). The Water Column Model included 13,129 observations, while the Bottom Conditions Model included 73,036 observations. Fewer observations were available for the Water Column Model as only locations between 10:00 and 14:00 and ≥ 50 m could be used so that chl-*a* could be included. Although grey seal movements were concentrated over the eastern Scotian Shelf and lower Gulf of St. Lawrence, as noted above (Fig. 1), ranges and movement patterns were quite variable among individuals (e.g., Fig. 4).

### HMM fitting

**Fig. 5:**
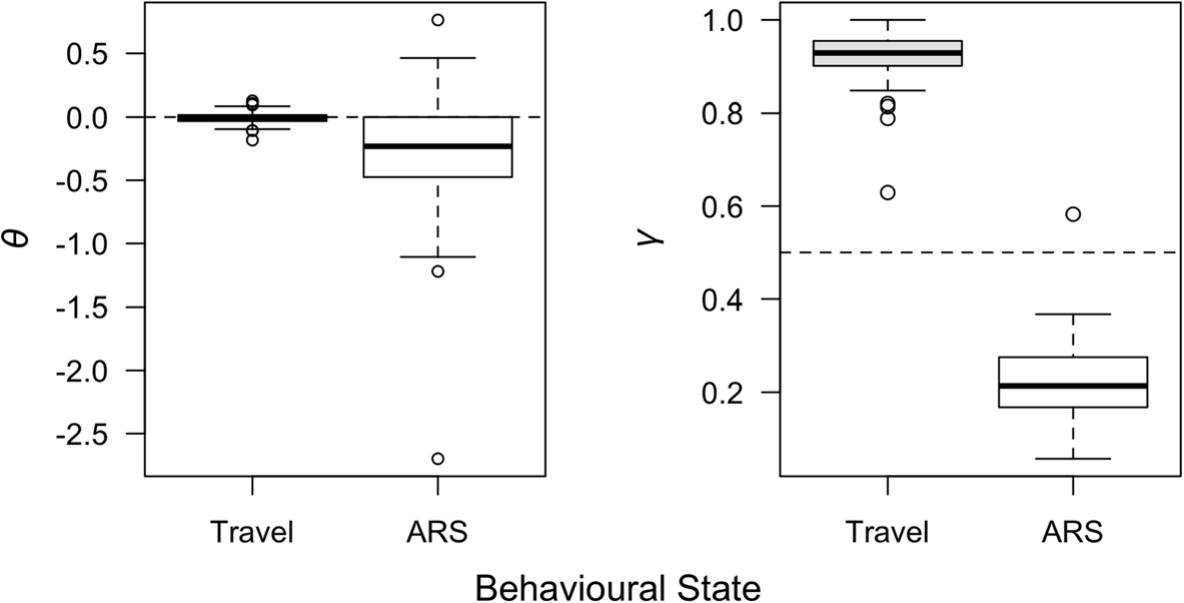
Parameter estimates of *θ* (i.e., turning angle) and *γ* (i.e., autocorrelation in both direction and speed) for each behavioural state estimated using hidden Markov models fitted with the R package *swim* [55]

HMMs estimated two distinct sets of parameters in all tracks (Fig. 5). Estimates of *θ* for the travelling behavioural state were closely centered around zero, corresponding well with persistent directional movements to foraging patches, evident in mapped behavioural states (Fig. 4). Estimates of *θ* for the apparent foraging behavioural state were transformed to center around zero for interpretation, because many of the output estimates were near multiples of 2π (i.e., a complete circle). Aside from a single outlier, *γ* estimates indicated distinct similar, faster movements (*γ* ≈ 0.9 – travelling) and dissimilar, slower movements (*γ* ≈ 0.2 – apparent foraging).

### Effects of covariates in Water Column Model

Estimates of the spatio-temporal distribution of chl-*a* from the bio-optical model [50] are illustrated for several individuals in Fig. 4. Season had no effect on the odds of observing apparent foraging, but females were three times more likely than males to be in the apparent foraging state at any given time (Table 3). There was no evidence for a sex-season interaction. Although *T*_*50*_ had no effect on foraging state in males, for every increase in 1.0 °C, females were 6.6% less likely to be foraging. There was no seasonal effect of *T*_*50*_ on the probability of apparent foraging. However, for every 1.0 mg m^− 3^ increase in chl-*a*, there was an almost 100% increase in odds of observing the apparent foraging state, in both males and females. Nevertheless, estimated variability in chl-*a* was relatively low (Table S2). The effect of chl-*a* on the odds of observing the apparent foraging state was about 70% less in summer than in fall.

**Table 3:** Water Column Model (Model 1) coefficients of the odds of being in the apparent foraging state. The intercept representing males in fall. Coefficients are exponentiated to odds ratios with upper and lower 95% confidence limits

	Coefficient (SE)	Lower	Odds Ratio	Upper	***P***-value
Intercept	−0.09 (0.30)	0.50	0.92	1.66	0.77
Season (Summer)	0.32 (0.36)	0.68	1.38	2.79	0.37
Sex (Female)	1.40 (0.36)	2.02	4.07	8.17	**< 0.001**
*T*_*50*_	0.02 (0.03)	0.96	1.01	1.07	0.59
Chl-*a*	0.68 (0.29)	1.12	1.98	3.48	**0.02**
Season (Summer): *T*_*50*_	−0.01 (0.03)	0.93	0.99	1.05	0.75
Season (Summer): Chl-*a*	−1.07 (0.33)	0.18	0.34	0.66	**< 0.01**
Sex (Female): *T*_*50*_	−0.07 (0.03)	0.88	0.93	1.00	**0.04**
Sex (Female): Chl-*a*	−0.30 (0.34)	0.38	0.74	1.44	0.38
Season (Summer): Sex (Female)	0.16 (0.20)	0.79	1.18	1.75	0.42

### Effects of covariates in Bottom Conditions Model

Season was not a significant predictor of behavioural state and there were no seasonal relationships with any properties associated with the bottom of dives (Table 4). As in the Water Column Model, there were sex-specific differences in the odds of observing the apparent foraging state, where females were over twice as likely as males to be foraging at any given time. There was also a significant sex-by-season interaction, whereby females in summer were about 50% more likely than males to be in the apparent foraging state. More time spent at the bottom of the dive reduced the odds of observing the apparent foraging state for males and females, however the effect was small and unlikely to be biologically significant in both cases. An increase in bottom duration of males by 1 s reduced the odds of foraging by about 0.13%, whereas in females, it was reduced by only 0.08%. The odds of observing the apparent foraging state increased by 2.1% with every 1 °C increase in bottom temperature for both sexes, but again, the effect was small. Dive depth did not significantly influence the behavioural states of males, however in females, deeper dives increased the odds of observing the travelling state. Every doubling of dive depth increased the odds of being in the travelling state by about 8.4%.

**Table 4:** Bottom Conditions Model (Model 2) coefficients of the odds of being in the apparent foraging state. The intercept represents males in fall. Bottom depth was log-transformed prior to model fitting. Coefficients are exponentiated to odds ratios with upper and lower 95% confidence limits

	Coefficient (SE)	Lower	Odds Ratio	Upper	***P***-value
Intercept	0.45 (0.24)	0.98	1.56	2.49	0.06
Season (Summer)	−0.10 (0.22)	0.59	0.91	1.39	0.65
Sex (Female)	1.20 (0.29)	1.90	3.33	5.86	**< 0.001**
Bottom Duration	−0.00 (0.00)	1.00	1.00	1.00	**< 0.001**
Bottom Temperature	0.02 (0.01)	1.01	1.02	1.04	**< 0.01**
Bottom Depth	0.02 (0.02)	0.98	1.02	1.05	0.34
Season (Summer): Duration	0.00 (0.00)	1.00	1.00	1.00	0.76
Season (Summer): Temperature	−0.00 (0.01)	0.98	1.00	1.01	0.63
Season (Summer): Depth	−0.06 (0.03)	0.89	0.94	1.00	0.05
Sex (Female): Duration	0.00 (0.00)	1.00	1.00	1.00	**< 0.01**
Sex (Female): Temperature	−0.01 (0.01)	0.98	0.99	1.01	0.59
Sex (Female): Depth	−0.09 (0.03)	0.87	0.92	0.96	**< 0.001**
Season (Summer): Sex (Female)	0.38 (0.11)	1.18	1.46	1.82	**< 0.001**

## Discussion

The results of this study provide evidence that grey seal foraging behaviour is associated with the fine-scale oceanographic conditions they encounter that presumably directly, or indirectly influence the distribution of prey across the SS ecosystem. Chl-*a* varied seasonally and was positively associated with observing apparent foraging behaviour, particularly during the fall phytoplankton bloom. Areas of increased primary productivity have also been shown to correlate with foraging behaviour in other pinniped species [30, 61]. In female grey seals, apparent foraging occurred more often in areas with cooler *T*_*50*_, which may be indicative of increased thermal stratification corresponding with preferred prey species [43, 62], which make vertical migrations, such as sand lance (*Ammodytes dubius*) or redfish (*Sebastes sp.*). The importance of water mass properties on prey species has also been observed in other pinnipeds [20]. In southern elephant seals, switching from the directed to the resident state is associated with cooler waters and increased thermal stratification [21]. Bottom temperature, dive depth, and bottom duration were all significantly related to the probability of observing apparent foraging behaviour, although the effects were small. As reported in other pinniped species [63, 64], our findings suggest that grey seals may exhibit preferences for the temperature and depth conditions preferred by prey species.

### Sex-specific, seasonal foraging behaviours

Sex-specific, seasonal differences in foraging effort were present among grey seals, as previously reported for this species [42, 65, 66]. The lack of a seasonal effect in the probability of apparent foraging in the Water Column Model could be the result of only including dives occurring ≥50 m and between the hours of 10:00 and 14:00 AST, greatly reducing the number of observations. By contrast, in the Bottom Conditions Model where more observations were available, seasonal and sex-specific differences in apparent foraging behaviour over the 7-month pre-breeding period were evident. Sex-specific foraging behaviours are thought to reflect differences in body energy storage and expenditures throughout the year, and are consistent with differences in the timing of mass gain and diet composition [33, 43, 62, 65]. The consequences of insufficient mass gain prior to the breeding season are higher for females than for males [67, 68], because heavier females produce larger pups at weaning which have improved chances of survival [69]. This may be why females were more likely to be observed in the apparent foraging state than males. Females generally consume a higher energy density and more specialized diet of smaller prey than males (e.g., sand lance), and thus may have to forage more often to satisfy their energy requirements [43]. Females were most likely to be in the apparent foraging state during summer, consistent with exploiting foraging patches closer to Sable Island, resulting in proportionally less time spent in the travelling state [33].

### Association of behavioural states with oceanographic conditions

The response variable in our GLMMs (i.e., behavioural states predicted from HMMs) was treated as known without error. We were unable to incorporate covariates directly into the transition probability estimation in the HMM because of the drastically different sampling scales for the oceanographic conditions. Although a two-stage analysis was the only way to feasibly test our hypothesis, we recognize that we were unable to account for error in HMM state prediction within the GLMM framework. Given the sample size and that HMM results demonstrated clear outbound, foraging, and inbound trip segments, consistent with previous movement analyses for this population [33], we have confidence in our use of HMM behavioural state predictions in the GLMMs.

We chose to fit a two-state model rather than a three-state model for comparison with previous research on our study population. We are aware that other research on pinniped foraging behaviour, including grey seals, has suggested that a two-state model may overlook resting or sleeping at sea, and as a consequence overestimate foraging (e.g., [66, 70, 71]). Animal-borne video and accelerometry data from adult grey seals in our study population also suggests that a two-state behavioural model overlooks resting at the surface and sleeping at depth [Lidgard, Broell, and Bowen unpublished], and therefore likely overestimates the time spent foraging reported in Table 2. Future studies may therefore benefit from attempting to estimate additional behavioural states from tracking data.

Chl-*a* data were estimated using the bio-optical model and included uncertainty that was not accounted for in subsequent modelling. Nonetheless, chl-*a* was a useful predictor of behavioural states indicating that grey seals generally exploit predictably productive areas on or near offshore banks. The behaviours of large marine predators of diverse taxa correspond with oceanographic features associated with increased primary productivity (e.g., [27, 29]). The association of apparent foraging with chl-*a* was greater in the fall, corresponding with the fall phytoplankton bloom when spatial variation in chl-*a* may be more heterogeneous [39]. Southern elephant seals (*Mirounga leonina*) have also shown seasonal associations of foraging behaviour with areas of high phytoplankton biomass related to bloom periods and the aggregation of lower-trophic level prey [72]. Together with observed variation in movement patterns between sexes and seasons (Fig. 2) and among individuals (Fig. 4) [73], these results suggest that oceanographic conditions may play a role in generating individual variability in diets that has been previously observed in grey seals [43]. Estimates of *γ* for apparent foraging behaviour showed higher variability, which may reflect differences in foraging behaviour among individuals. While the shelf-slope front is largely outside of the grey seal habitat, at least one male exhibited movement patterns corresponding to the position of the shelf-break and shelf-slope front (Fig. 1), an area which is known to be highly productive [74]. In previous deployments during these periods, males showed a higher association with this area than seen here [34].

The results of the Water Column Model indicate that females apparently forage in areas with cooler *T*_*50*_. This corresponds well with their overall habitat distribution, as movements made by females were concentrated over the eastern Scotian Shelf and lower Gulf of St. Lawrence, in contrast to males which were more widespread across the region. These waters are stratified due to density gradients, with a cooler, fresher layer originating from the Gulf of St. Lawrence and a warmer, more saline bottom layer originating from the shelf-slope; in summer, heating of the upper layer results in an additional warm surface layer [38]. In other pinniped species, such as the northern fur seal (*Callorhinus ursinus*), foraging is more prevalent in areas with strong thermoclines [17]. When stratification of SS waters is high, phytoplankton become concentrated at or above the mixed layer depth and the vertical distribution of zooplankton (e.g., *Calanus finmarchicus*) closely follows [75]. Prey species which make diel vertical migrations to forage, such as sand lance, should respond to the availability of resources within the water column. Female grey seals have shown both diel variability in dive depths associated with movement of prey within the water column [42] and a dietary niche indicating that they consume a higher proportion of prey species that forage pelagically [43, 62]. While this provides a potential explanation for these results, it is possible that a combination of other factors may be influential (e.g., relationship between *T*_*50*_ and chl-*a* [51]).

Bottom temperature has been shown to influence both dive properties [63] and habitat use [64] corresponding to foraging in other pinniped species. Warmer bottom temperatures were associated with apparent foraging in grey seals, regardless of either sex or season. As exothermic species in this region have been found to migrate to warmer, shallower banks during summer and fall [76, 77] it is possible that grey seals are following these temperature-keeping species. This highlights the value of collecting in situ oceanographic measurements that are relevant to the conditions that grey seals encounter. Grey seals may perhaps be altering their foraging patterns to follow both the temperature and depth preferences of their prey species as distributions shift throughout the seasons [33, 77]. This would provide an explanation for the seasonal variability in the distributions of grey seals [34] and lack of seasonal interaction for oceanographic properties that were otherwise associated with behavioural state.

To increase the net energy gained during foraging trips, animals should only dive as deep as necessary to encounter prey and should maximize time spent at the bottom of the dive [78]. Variability in bottom time was large across states, sexes, and seasons (Table S2). However, females were more likely to perform shallower dives, which may allow them to maximize time spent at the bottom of the dive where prey are more likely to be encountered. This is consistent with a previous finding that females spend more time at the bottom of dives than males [42] and exhibit apparent foraging in areas of shallower bathymetry [42]. As bottom duration increased, both females and males were more likely to be in the travelling state. This is not entirely unsurprising as dive depth did not differ between apparent foraging and travelling states and, as noted above, may reflect higher energy expenditure during prey capture. Given the mean duration and variation in bottom times (Table S2), the number of dives made per day (Table 2), and the proportion of time at sea spent foraging (Table 2; [42]), the relatively small differences reported here could become biologically important. As almost all grey seal dives occur in bouts [65, 73], the use of a three-hour time step may have masked some variation in dive duration or depth. This supports previous findings that environmental variables may become important at some scales and not others [73, 79]. Given our current understanding of sex-specific differences in dive behaviours at early developmental stages [80] it is possible that these sex-specific relationships with oceanographic conditions develop early in life and may persist into reproductive age. Grey seals of both sexes dive to depth during both foraging and travelling dives. Whether this is solely an evolutionary adaptation for predator avoidance during travelling [81, 82], to increase opportunistic prey encounters [73], or a mechanism for encountering suitable conditions for foraging habitat is beyond the scope of this study.

### Fine-scale habitat use

The high-resolution GPS locations obtained within this study revealed the fine-scale nature of habitat use by grey seals (Fig. 3). Although it has been previously noted that habitat boundaries of grey seals seem well-defined over shallow banks [33], the way in which these topographical features are used was much more precise than anticipated. For example, Middle Bank (44°50′N, 60°50′W) has been long regarded as a foraging hotspot for this population [33, 34]. This area is ecologically important as a source of primary productivity and for its high fish species richness [83]. It has also been regarded as a major spawning habitat for sand lance [84]. Our results show that, aside from a small area at the western boundary, grey seals almost exclusively used the eastern half of Middle Bank, which provides evidence of the fine-scale nature of grey seal movements (Fig. 3). This is particularly interesting, as this pattern of space-use was consistent across all individuals sampled from this population. Whether this is attributable to prey preferences for bottom temperature or depth, seabed morphology and substrate, circulation patterns, or some combination remains to be seen. French Bank, located nearby to Middle Bank, showed a similar pattern of specific partial-use by grey seals (Fig. 3). By contrast, essentially all of Canso Bank (45°20′N, 60°30′W) was heavily used by grey seals (Fig. 3). Reliance upon Canso Bank by males and females in both seasons sampled during the study period corresponds well with the high abundance of sand lance [83] and prevalence of this species in the grey seal diet [43]. These results provide further evidence that while bathymetric features may provide suitable habitat for grey seal prey species, the complexity of oceanographic processes are clearly influential on the movements of grey seals across the continental shelf. Interpretation of these movement patterns may benefit from further investigation of the oceanographic conditions associated with habitat use and availability. The fine-scale habitat selection by this large marine predator also underscores the difficulty in using broad-scale overlap of prey distributions as the basis for inferences about predation and mortality.

## Conclusions

Our study shows that oceanographic conditions encountered by grey seals during the course of foraging trips, which may directly or indirectly structure the prey field, were associated with estimated at-sea behavioural states. In the Water Column Model, chl-*a* was a useful predictor of foraging behaviour, together with upper-water column temperature, and sex. Whereas in the Bottom Conditions Model, although the odds of females foraging was more than twice that of males, oceanographic conditions measured had only a small association with behaviour. Season alone had no effect on the probability of observing apparent foraging in either model. Our results demonstrate the value of using high resolution oceanographic data collected from instrumented animals at scales relevant to foraging decisions made by large marine predators. Visualization of fine-scale location data demonstrated the highly specific nature of habitat use, highlighting the importance of considering how other oceanographic processes may shape the foraging distributions of grey seals and other marine species.

## Supplementary information

**Additional file 1: Table S1.** Sample means and standard deviations (SD) of age, body mass, and body length data collected for instrumented grey seals (*n* = 79). Between 1969 and 2002, groups of female and male grey seals were branded at weaning, producing a pool of individually identifiable, known-age adults [45]. Individuals were selected from this pool in addition to nine unbranded adults. Once immobilized, grey seals were weighed using a 300 kg (±1 kg) Salter spring balance (2009 to 2012) or a 500 kg (± 1 kg) Tractel (www.tractel.com) load cell (2013 to 2015); standard body length was also taken at this time. **Table S2.** Sample means and standard deviations (SD) of oceanographic properties measured by grey seals (*n* = 79) used in the Water Column Model (Model 1) and Bottom Conditions Model (Model 2), including chlorophyll-*a* concentration (chl-*a*; mg m^− 3^), upper-water column temperature (*T*_*50*_; °C), bottom temperature (°C), bottom depth (m), and bottom duration (s); values are separated by season, sex, and behavioural states estimated by hidden Markov models.

## Data Availability

Data from this study supporting the conclusions of this article are archived with the Ocean Tracking Network (http://oceantrackingnetwork.org/). Access to these data is available from the corresponding author on reasonable request.

## Abbreviations

Chl-*a*: Chlorophyll-*a* concentration
GLMM: Generalized linear mixed-effects model
HMM: Hidden Markov Model
*LA*: Light attenuation
*LL*: Light level
SS: Scotian Shelf
TDLR: Time-Depth-Light Recorder
*T*_*50*_: Mean upper-water column temperature
