# Supplementary material for: Foraging behaviour of a continental shelf marine predator, the grey seal (Halichoerus grypus), is associated with in situ, subsurface oceanographic conditions
Source: Mov Ecol. 2020 Oct 20;8:41. doi: 10.1186/s40462-020-00225-7 (PMC7574573; doi:10.1186/s40462-020-00225-7)
Supplement: Supplementary file 1 — Additional file 1: Table S1. Sample means and standard deviations (SD) of age, body mass, and body length data collected for instrumented grey seals (n = 79). Between 1969 and 2002, groups of female and male grey seals were branded at weaning, producing a pool of individually identifiable, known-age adults [45]. Individuals were selected from this pool in addition to nine unbranded adults. Once immobilized, grey seals were weighed using a 300 kg (±1 kg) Salter spring balance (2009 to 2012) or a 500 kg (± 1 kg) Tractel (www.tractel.com) load cell (2013 to 2015); standard body length was also taken at this time. Table S2. Sample means and standard deviations (SD) of oceanographic properties measured by grey seals (n = 79) used in the Water Column Model (Model 1) and Bottom Conditions Model (Model 2), including chlorophyll-a concentration (chl-a; mg m− 3), upper-water column temperature (T50; °C), bottom temperature (°C), bottom depth (m), and bottom duration (s); values are separated by season, sex, and behavioural states estimated by hidden Markov models. [file 40462_2020_225_MOESM1_ESM.docx]

**Supplementary Material**

|  | **2009** | | **2010** | | **2011** | **2013** | | **2014** | | **2015** |
| --- | --- | --- | --- | --- | --- | --- | --- | --- | --- | --- |
|  | **M** | **F** | **M** | **F** | **F** | **M** | **F** | **M** | **F** | **F** |
| **Age** | 21.0 | 22.5 | 16.7 | 23.9 | 24.3 |  | 24.7 | 14.2 | 25.7 | 28.1 |
| SD | 5.61 | 1.60 | 8.12 | 1.41 | 1.70 |  | 5.22 | 1.48 | 4.92 | 1.69 |
| **Mass** | 258.1 | 176.6 |  | 170.0 | 208.7 | 282.3 | 193.4 | 292.0 | 199.0 | 215.4 |
| SD | 28.70 | 23.95 |  | 1.41 | 6.51 | 31.35 | 30.32 | 29.11 | 27.25 | 28.71 |
| **Length** | 214.4 | 188.5 | 208.0 | 186.4 | 184.8 | 211.0 | 188.87 | 206.2 | 181.3 | 186.8 |
| SD | 5.77 | 5.21 | 14.20 | 7.83 | 7.16 | 6.78 | 4.13 | 6.61 | 5.62 | 10.23 |

Table S1. Sample means and standard deviations (SD) of age, body mass, and body length data collected for instrumented grey seals (n = 79). Between 1969 and 2002, groups of female and male grey seals were branded at weaning, producing a pool of individually identifiable, known-age adults (Lidgard et al. 2014). Individuals were selected from this pool in addition to nine unbranded adults. Once immobilized, grey seals were weighed using a 300 kg (±1 kg) Salter spring balance (2009 to 2012) or a 500 kg (± 1 kg) Tractel (www.tractel.com) load cell (2013 to 2015); standard body length was also taken at this time.

36

|  | **Travelling** | | | | **Foraging** | | | |
| --- | --- | --- | --- | --- | --- | --- | --- | --- |
|  | **Male** | | **Female** | | **Male** | | **Female** | |
|  | **Summer** | **Fall** | **Summer** | **Fall** | **Summer** | **Fall** | **Summer** | **Fall** |
| **Chl-*a*** | 0.52 | 0.39 | 0.41 | 0.36 | 0.54 | 0.42 | 0.39 | 0.38 |
| SD | 0.16 | 0.23 | 0.19 | 0.20 | 0.19 | 0.22 | 0.16 | 0.20 |
| ***T_50_*** | 9.38 | 8.77 | 8.78 | 9.28 | 8.84 | 9.02 | 8.68 | 9.06 |
| SD | 2.11 | 2.37 | 2.25 | 2.66 | 1.76 | 2.36 | 2.28 | 2.44 |
| **Bottom Temperature** | 5.42 | 6.50 | 5.35 | 6.07 | 5.34 | 6.50 | 6.05 | 5.77 |
| SD | 4.12 | 3.52 | 3.24 | 3.08 | 4.86 | 4.00 | 4.33 | 3.32 |
| **Bottom Depth** | 59.63 | 63.21 | 67.32 | 65.29 | 60.42 | 60.44 | 63.90 | 64.17 |
| SD | 35.76 | 39.78 | 39.56 | 37.74 | 37.24 | 39.14 | 36.38 | 33.57 |
| **Bottom Duration** | 209.16 | 211.47 | 303.35 | 269.41 | 216.17 | 198.95 | 297.63 | 259.29 |
| SD | 95.40 | 89.61 | 142.47 | 111.41 | 104.33 | 95.87 | 154.17 | 120.67 |

37

Table S2. Sample means and standard deviations (SD) of oceanographic properties measured by grey seals (n = 79) used in the Water Column Model (Model 1) and Bottom Conditions Model (Model 2), including chlorophyll-*a* concentration (chl-*a*; mg m^-3^), upper-water column temperature (*T_50_*; ºC), bottom temperature (ºC), bottom depth (m), and bottom duration (s); values are separated by season, sex, and behavioural states estimated by hidden Markov models.
